# Goldmann applanation tonometry compared with corneal-compensated intraocular pressure in the evaluation of primary open-angle Glaucoma

**DOI:** 10.1186/1471-2415-12-52

**Published:** 2012-09-25

**Authors:** Joshua R Ehrlich, Nathan M Radcliffe, Mitsugu Shimmyo

**Affiliations:** 1 Department of Ophthalmology, Weill Cornell Medical College, 1305 York Avenue, New York, NY, 10021, USA; 2 New York Eye and Ear Infirmary, Department of Ophthalmology, New York Medical College, Valhalla, NY, USA

**Keywords:** Open-angle glaucoma, Low tension glaucoma, Intraocular pressure, Intraocular pressure, Ocular tonometry

## Abstract

**Background:**

To better understand the role of corneal properties and intraocular pressure (IOP) in the evaluation of primary open-angle glaucoma (POAG); and to determine the feasibility of identifying glaucomatous optic neuropathy (GON) using IOP corrected and uncorrected for corneal biomechanics.

**Methods:**

Records from 1,875 eyes of consecutively evaluated new patients were reviewed. Eyes were excluded if central corneal thickness (CCT) or Ocular Response Analyzer (ORA) measurements were unavailable. Presence or absence of GON was determined based on morphology of the optic disc, rim and retinal nerve fiber layer at the time of clinical examination, fundus photography and Heidelberg Retinal Tomography. Goldmann-applanation tonometry (GAT) in the untreated state was recorded and Goldmann-correlated (IOPg) and corneal-compensated IOP (IOPcc) were obtained using the ORA. Glaucomatous eyes were classified as normal or high-tension (NTG, HTG) using the conventional cutoff of 21 mm Hg. One eligible eye was randomly selected from each patient for inclusion.

**Results:**

A total of 357 normal, 155 HTG and 102 NTG eyes were included. Among NTG eyes, IOPcc was greater than GAT (19.8 and 14.4 mm Hg; p < 0.001) and the difference between IOPcc and GAT was greatest for this subgroup of patients with NTG (p ≤ 0.01). The maximum combined sensitivity and specificity for detection of GON occurred at 20.9 mm Hg for GAT (59%, 90%) and 18.4 mm Hg for IOPcc (85%, 85%) and the area under the curve was greater for IOPcc (0.93 vs. 0.78; p < 0.001).

**Conclusions:**

IOPcc may account for measurement error induced by corneal biomechanics. Compared to GAT, IOPcc may be a superior test in the evaluation of glaucoma but is unlikely to represent an effective diagnostic test.

## Background

Glaucoma is an optic neuropathy for which intraocular pressure (IOP) is the only known modifiable risk factor. Historically, elevated IOP was essential to the diagnosis of glaucoma, however the current American Academy of Ophthalmology definition does not include elevated IOP as a requirement [1,2]. IOP as measured by Goldmann applanation tonometry (GAT) does not correlate well with glaucomatous optic neuropathy (GON) as there are entities such as ocular hypertensive eyes with elevated GAT readings without GON as well as normal tension glaucoma (NTG) eyes with progressive GON despite normal or low GAT measurements [3-6].

Notwithstanding, data from clinical trials support a role for IOP reduction in patients with ocular hypertension, early or advanced primary open-angle glaucoma (POAG), and NTG [7-11]. As IOP remains the only modifiable risk factor for eyes with GON and IOP reduction remains the mainstay of treatment, greater accuracy and less confounding in IOP measurement should have value in the management of most glaucomas [7-11]. It is not entirely clear whether the inconsistent association between IOP and glaucoma is related to shortcomings of office based IOP measurement, or whether IOP-independent risk factors are responsible for the pathological process in some glaucomas. While both may be operative, there is evidence that in applanation tonometry various factors can cause systematic measurement errors. In fact, Goldmann warned that extremely thick corneas would be measured inaccurately [12]. Ehlers measured IOP by in vivo cannulation of human eyes and introduced a table to correct the GAT errors caused by variations in central corneal thickness (CCT) measured by optical pachymetry [13]. Liu and Roberts quantitatively analyzed the theoretically larger effect of biomechanical properties of the cornea in addition to corneal curvature and thickness [14]. Also, recent studies have shown that GAT calibration error is common [15].

The Ocular Response Analyzer (ORA; Reichert Ophthalmic Instruments, Buffalo, NY) functions as a non-contact tonometer and was designed to provide IOP assessments that are independent of CCT. The instrument provides a Goldmann-correlated measure of IOP (IOPg) and a corneal compensated IOP (IOPcc) that is not correlated with CCT [16]. A number of investigators have illustrated that the ORA may avoid some of the aforementioned drawbacks of GAT, including confounding by corneal thickness, calibration error, and concerns regarding contamination and sanitation [17-21]. It has been demonstrated that IOPg serves as a good approximation of GAT and may offer the clinician a tool to transition toward a corneal compensated IOP without fully abandoning GAT [17,22]. Ultimately, transitioning to a corneal compensated measure of IOP might provide the clinician with a better approximation of anterior chamber pressure, independent of corneal biomechanical properties [18,21,23,24].

It is unclear whether an IOP estimation less affected by confounding corneal properties could more effectively detect and/or aid in the evaluation and management of glaucoma. In the present study we sought to compare the sensitivity and specificity of measurements corrected and uncorrected for corneal biomechanics for the detection of GON in a population of patients under evaluation in a comprehensive ophthalmology office.

## Methods

Data collection for this cross-sectional retrospective study was performed within the private office of one of the authors (MS). Approval for this cross-sectional retrospective study was obtained from the institutional review board at the New York Eye and Ear Infirmary and this investigation adhered to the tenets of the Declaration of Helsinki.

We reviewed IOP measurements for 1,875 consecutive eyes of new patients evaluated from January 2, 2004 to December 31, 2007 in a comprehensive ophthalmology practice in New York City. Consecutive patients with or without glaucoma undergoing initial eye examination were included in the study. Eyes were classified as normal or glaucomatous based on the absence or presence of morphological GON ascertained by clinical examination, fundus photography and Heidelberg Retinal Tomography II (Heidelberg Engineering, Heidelberg, Germany) of the optic disc and neuroretinal rim using previously defined criteria [25] that include changes such as: optic disc asymmetry, vertical/horizontal disparity, rim area and rim volume asymmetry, notching of the rim, violation of the ISNT rule and disc hemorrhages, with or without visual field defects, regardless of IOP level.

Eyes of patients without clinically evident GON were designated as a normal group, including patients under evaluation for glaucoma who were generally referred on the basis on family history or physiologic cupping.

All included patients had undergone CCT measurements with the DGH 550 pachette ultrasonic pachymeter (DGH Technologies, Pensylvania) and evaluation with the ORA (Reichert Inc, Depew, NY). Eyes without documented CCT or ORA measurements were excluded. All patients in this practice underwent routine CCT and ORA assessment regardless of diagnosis or reason for referral; however these were not always performed on the same visit and patients were only included when these data were acquired on the same date. Other exclusion criteria included acute or chronic angle closure glaucoma; secondary glaucoma; pseudoexfoliation syndrome; eyes with previous medical, laser or surgical treatment for glaucoma or corneal conditions; and lens and vitreous opacities that prevented optic nerve and retinal nerve fiber examinations. In addition, eyes were excluded if there was a history or diagnosis of ischemic optic neuropathy, vascular occlusive diseases, cerebrovascular accident, or other pertinent neurological disease. Data were extracted for analysis by randomly selecting one eye of each subject.

The highest GAT value recorded in an eye in the untreated state (no ocular hypotensive medication) was included for analysis. Topical anesthetic and fluorescein was placed in each eye before GAT, which was performed immediately after ORA evaluation per office protocol. The GAT used for the study was checked monthly to ensure proper calibration. Additionally, IOPg and IOPcc values were collected from the ORA. Per office protocol, ORA measurements were repeated five times or until a waveform score of 6.5 was obtained, and the measurement with the best waveform score was selected for inclusion in the study.

Using the conventional delineating GAT value of 21 mm Hg, we divided the population of all glaucomatous eyes into 2 groups: eyes with GAT greater than 21 mm Hg were designated as high-tension glaucoma (HTG); and eyes with GAT of 21 mm Hg or less as NTG. NTG eyes were then subdivided by IOPcc values; IOPcc greater than 21 mm Hg (high IOPcc NTG) and IOPcc less than or equal to 21 mm Hg (low IOPcc NTG). In each category and subgroup, age, GAT, IOPg and IOPcc were compared.

Differences in age, GAT, IOPg and IOPcc were determined among normal, HTG and NTG eyes. Sensitivity and specificity of GAT and IOPcc for the detection of GON at different thresholds of IOP were determined from the entire study population of normal and glaucomatous eyes; presence or absence of GON, as previously described, was designated as the gold standard for comparison. Receiver operating characteristic (ROC) curves of GAT and IOPcc were then constructed. The optimum IOP cutoff was defined as the value corresponding to the point on the ROC curve closest to the upper-left corner. A multiple ANOVA and post-hoc analyses were also performed and Benferroni corrections were applied when indicated. All statistical tests were two-sided with a 0.05 level of significance.

## Results

A total of 1,261 normal eyes were excluded due to absence of CCT or ORA measurements. Demographic and IOP data for normal, HTG and NTG eyes are shown in Table 1 for the 614 patients included in the study (357 normal, 155 POAG HTG, 102 POAG NTG).

**Table 1:** Patient characteristics

**Category**	**Normal**	**HTG**	**NTG**
Criteria	No GON	GON + GAT > 21 mm Hg	GON + GAT ≤ 21 mm Hg
Number of patients/eyes	357	155	102
Age (years), mean ± SD	62.3 ± 13.4	66.3 ± 12.7*	68.7 ± 10.9*
GAT (mm Hg)	14.0 ± 2.4	25.3 ± 5.6*＾	14.4 ± 3.4＾
IOPg (mm Hg)	14.0 ± 3.0	26.5 ± 5.5*＾	14.4 ± 3.4＾
IOPcc (mm Hg)	14.5 ± 3.1	25.3 ± 5.5*＾	19.8 ± 3.4*＾
IOPcc-GAT (mm Hg)	0.5 ± 1.7	1.2 ± 1.2*＾	5.4 ± 1.1*＾
CCT (μm)	542 ± 33	553 ± 43*＾	513 ± 39*＾
Female	57%	63%	52%
Right eye	50%	50%	42%
Asian	23%	15%*	11%*
Caucasian	28%	5%*＾	26%＾
Mixed	47%	78%*＾	47%＾
Black	3%	3%＾	12%*＾

*p ≤ 0.05 for comparison with normal group.

＾p ≤ 0.05 for comparison between NTG and HTG groups.

HTG: high-tension glaucoma; NTG: normal-tension glaucoma; GON: glaucomatous optic neuropathy; GAT: Goldmann applanation tonometry; IOPg: Goldman-correlated intraocular pressure; IOPcc: corneal compensated intraocular pressure; CCT: central corneal thickness.

**Figure 1:**
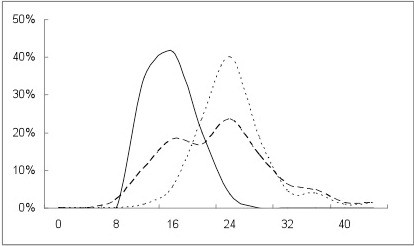
**Frequency distributions of IOP measurements in normal and glaucomatous eyes.** Frequency distribution of GAT in normal (solid line); GAT in glaucoma (dashed line); and IOPcc in glaucoma (fine dotted line); p < 0.001 for each pair-wise comparison. GAT, IOPg and IOPcc of normal eyes were statistically similar (p > 0.05), and are therefore represented by GAT (solid line).

Frequency distributions of IOP measurements in normal and glaucomatous eyes are shown in Figure 1. GAT was chosen to represent IOP in normal eyes since all pressure measurements (GAT, IOPg and IOPcc) were statistically equivalent in this group (p > 0.05). The frequency distribution of both GAT and IOPcc is illustrated for all glaucomatous eyes (Figure 1).

Among NTG eyes, mean IOPcc was significantly higher than mean GAT (19.8 vs. 14.4 mm Hg; p < 0.001). Furthermore, the difference between GAT and IOPcc (expressed as [IOPcc – GAT] ) was significantly greater in NTG (5.4 ± 1.1 mm Hg) than in either normal (0.5 ± 1.7 mm Hg; p < 0.001) or HTG eyes (1.2 ± 1.2 mm Hg; p < 0.001). CCT was significantly correlated with IOPcc in NTG (p = 0.01) but not normal (p = 0.48) or HTG eyes (p = 0.93).

In this study, NTG eyes were defined as eyes with GON and a maximum recorded GAT less than or equal to 21 mm Hg. However, 39 (38%) of the 102 GAT defined NTG eyes had an IOPcc higher than 21 mm Hg; the mean IOPcc of these eyes was 23.3 mm Hg (Table 2). Of the same cohort of NTG eyes, 73 (72%) had an IOPcc greater than 18 mm Hg, 93 (91%) had an IOPcc greater than 15 mm Hg, and only 9 eyes (9%) had an IOPcc of 15 mm Hg or less.

**Table 2:** Normal-tension glaucoma patient characteristics

**Category**	**All NTG**	**NTG with high IOPcc**	**NTG with low IOPcc**
Criteria	GAT ≤ 21 mm Hg	GAT ≤ 21 mm Hg, IOPcc > 21 mm Hg	GAT ≤ 21 mm Hg, IOPcc ≤ 21
Number of patients/eyes	102	39	63
Age (years), mean ± SD	68.7 ± 10.9	70.0 ± 8.6*	67.8 ± 12.1*
GAT (mm Hg)	14.4 ± 3.4	17.6 ± 1.4*	12.4 ± 2.6*
IOPg (mm Hg)	14.4 ± 3.4	17.6 ± 1.4*	12.4 ± 2.6*
IOPcc (mm Hg)	19.8 ± 3.4	23.3 ± 1.4*	17.7 ± 2.4*
IOPcc-GAT (mm Hg)	5.4 ± 1.1	5.6 ± 1.0	5.2 ± 1.1
CCT (μm)	513 ± 39	522 ± 35	507 ± 40
Female	52%	64%	46%
Right eye	42%	33%	49%
Asian	11%	8%	13%
Caucasian	26%	28%	25%
Mixed	47%	56%	41%
Black	12%	8%	16%

*p ≤ 0.05 for comparison between NTG with high IOPcc and NTG with low IOPcc.

HTG: high-tension glaucoma; NTG: normal-tension glaucoma; GON: glaucomatous optic neuropathy; GAT: Goldmann applanation tonometry; IOPg: Goldman-correlated intraocular pressure; IOPcc: corneal compensated intraocular pressure; CCT: central corneal thickness.

**Figure 2:**
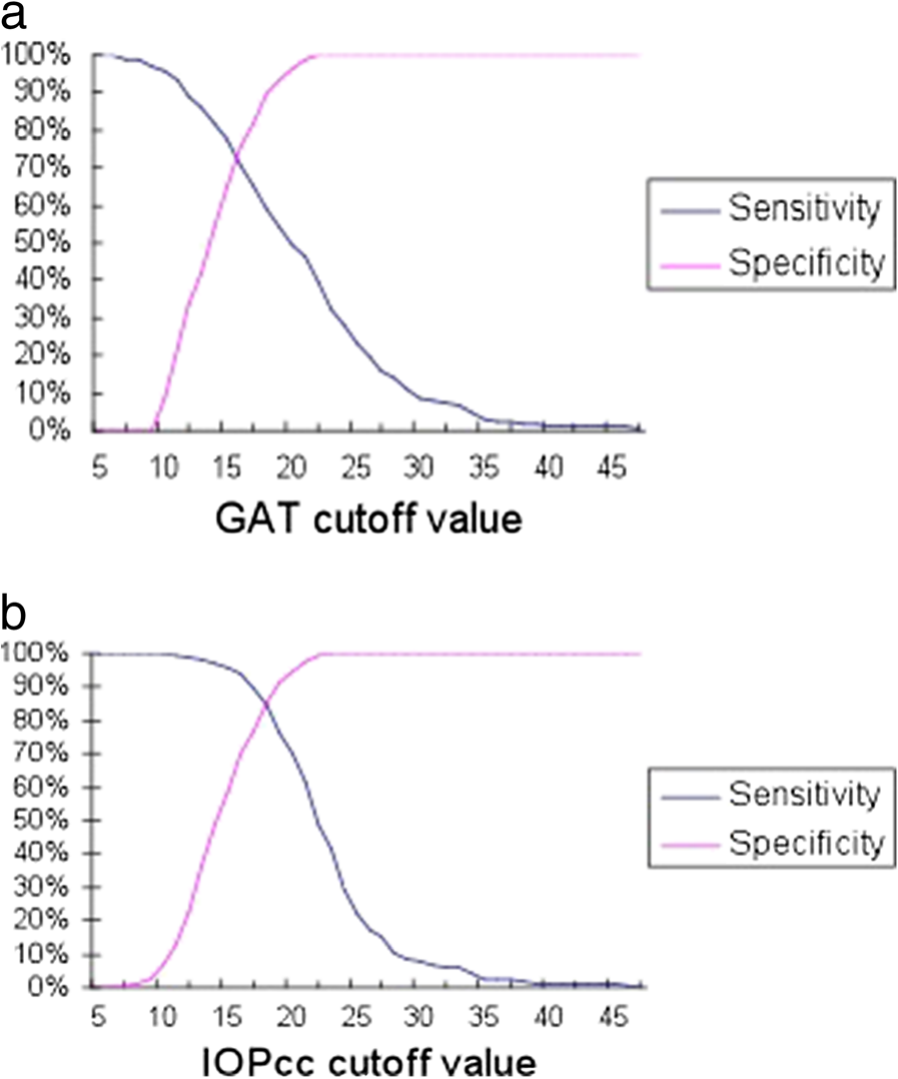
**Sensitivity and specificity of GAT and IOPcc in detecting glaucomatous optic neuropathy.a**. Sensitivity and specificity of GAT in detecting glaucoma. **b**. Sensitivity and specificity of IOPcc in detecting glaucoma.

**Figure 3:**
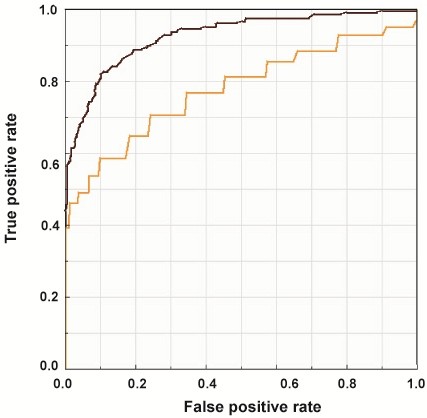
**ROC curves for GAT and IOPcc.** ROC curves of GAT and IOPcc in the detection of GON (orange line-GAT; black line-IOPcc). The AUC for GAT was 0.78 and for IOPcc was 0.93 (test for difference of AUCs: p < 0.001).

Sensitivities and specificities for identifying glaucoma using GAT (Figure 2a) and IOPcc (Figure 2b) were determined from the total study population of normal and glaucomatous eyes, using assessment of GON as the gold standard reference. The traditional GAT threshold of 21 mm Hg had a sensitivity of 46% (specificity 99%) for detecting glaucoma. The optimal GAT threshold to maximize combined sensitivity and specificity was 20.9 mm Hg (sensitivity 59%, specificity 90%). Likewise, an IOPcc cutoff of 18.4 mm Hg resulted in the highest combination of sensitivity (85%) and specificity (85%) for detecting glaucoma. The areas under the curve (AUC) of ROC curves were compared and demonstrated a greater AUC for IOPcc (0.93) compared to GAT (0.78) in the detection of GON (test for difference of AUCs: p < 0.001) Figure 3.

## Discussion

In this comparison of several different assessments of IOP for the detection of GON, we found evidence that an IOP assessment that compensates for corneal biomechanical properties may have better accuracy for the detection of POAG than GAT. Specifically, we found that GAT and IOPcc showed considerable agreement within the normal and HTG cohorts. However, among NTG eyes IOPcc was, on average, significantly higher than GAT. Moreover, we determined that the optimal threshold for GAT to maximize sensitivity and specificity for the detection of GON was 20.9 mm Hg and for IOPcc was 18.4 mm Hg. Using ROC curves we found that the AUC for the IOPcc (0.93) was greater than for GAT (0.78), indicating that compared to GAT, IOPcc may represent a superior test in the evaluation of glaucoma.

Importantly, we would like to emphasize that we do not support the use of even a corneal compensated IOP for the diagnosis of glaucoma. Due to the relatively low prevalence of POAG in the population [26-29], a diagnostic test with a sensitivity and specificity of 85% would result in low positive predictive value and thus a high number of false-positives. For example, in a population with a 4% prevalence of undiagnosed POAG, only about 19% of patients with a positive result would have the disease. Additionally, with a sensitivity of 85%, approximately one in six patients with POAG would not be detected. Notwithstanding, IOPcc may represent a better tool for the evaluation and management of POAG due to its more consistent association with the disease across a wide range of IOPs.

Since IOPcc was greater than GAT in majority of NTG eyes (92%) in this study, segregating NTG and HTG using a GAT threshold of 21 mm Hg may not be optimal. Other studies have also demonstrated an underestimation of IOP by applanation tonometry after correcting IOP for corneal properties [18,21,30-33]. The present study, like others, found that eyes with NTG had significantly thinner corneas [34,35], while patients with HTG had greater CCT compared to normals. Of note, one study, the Low Tension Glaucoma Study, did not find abnormally low CCT in a cohort of NTG patients [36]. However, the current study suggests that the impact of corneal properties on IOP determination may be greatest in eyes with NTG.

Past investigations have demonstrated that IOPcc is less dependent on corneal properties than traditional contact tonometry [18,23]. Medeiros and Weinreb found that CCT was significantly correlated with GAT but not IOPcc in a population under evaluation for glaucoma. Additionally, in the same study they found that the difference between GAT and IOPcc increased linearly as a function of increasing CCT [18]. The results of the present investigation are similar in that we observed no significant correlation between CCT and IOPcc among HTG and normal eyes. However, we did determine a significant correlation between CCT and IOPcc among eyes with NTG. This pattern may exist since lower IOP values have a greater biologically plausible range for upward adjustment due to CCT than do higher IOP values as a function of regression toward the mean. This finding suggests that an accurate algorithm to correct for the impact of CCT and/or corneal biomechanics on IOP is likely to be non-linear.

CCT is lowest in eyes with NTG [34,35] and low CCT may be both an independent risk factor for glaucoma progression [8] as well as a source of IOP measurement error [30-33]. Consequently, use of a corneal compensated IOP may be preferable to GAT specifically in the evaluation and management of NTG. The existence of higher than normal IOPcc in eyes with NTG is supported by reports that medical and surgical therapies that decrease IOP in NTG eyes are effective in reducing the progression of GON [10,11]. In the Collaborative Normal Tension Glaucoma Treatment Study [10], the average baseline IOP in the treated and untreated arms was 16.1 and 16.9 mm Hg, respectively. With treatment, the average IOP in the treatment group was lowered to 10.6 mm Hg, while IOP in the control group remained 16.0 mm Hg. Importantly, 35% of untreated eyes and only 12% of treated eyes met criteria for visual field or glaucomatous optic disc progression at the end of the study [10].

Notwithstanding, it has been observed that some eyes with NTG progress despite ocular hypotensive therapy [10]. In fact, some have speculated that NTG eyes may even develop GON independent of IOP [37]. Given the observation in the present study that differences in IOPcc and GAT in a single patient may be as large as 8 mm Hg, an eye with a GAT value in the normal range may actually have a much higher IOPcc, depending on its corneal properties. In this context, setting a therapeutic target IOP using GAT would similarly differ from an IOPcc target pressure. This may partially explain glaucoma progression in NTG eyes in which IOP, as measured by GAT, appears to be adequately controlled since determining a safe IOP target is made difficult.

There are several limitations to this study. As an observational cross-sectional study, it is not possible to determine how the longitudinal risk of glaucoma progression is related to GAT compared to IOPcc. The operational definition of GON used in this study was based on the appearance of the optic nerve as documented by the evaluating clinician and data for other definitions of glaucoma including visual field defects are not available. While perimetric evaluation or other computerized optic nerve imaging techniques could have been employed, these devices have limitations related to accuracy for GON detection [38] and such testing was not performed on all patients in this study. Additionally, since CCT [30] and CH [39,40] differ by race/ethnicity it is possible that studies with distinct ethnic compositions may yield different results. Nonetheless, we did not separately analyze racial/ethnic differences in CCT and their contribution to our results. Finally, it was outside of the scope of this study to correlate the degree of GON with IOP levels or their fluctuation. Though we recognize that the absence of diurnal IOP data may have resulted in misclassification of some patients, there is no reason to believe that the data were systematically biased toward IOP measurement at any specific time of day.

This study contributes to the understanding of differences that exist in the impact of corneal properties on IOP measurement between normal, HTG and NTG eyes. Importantly, these results suggest that the a corneal compensated IOP may offer an attractive alternative to GAT, particularly for the evaluation and management of patients with NTG in whom corneal biomechanics and thickness appear to play the greatest role.

## Conclusions

The sensitivity and specificity of GAT and corneal-compensated IOPcc for the identification of GON were optimized at thresholds of 20.9 and 18.4 mm Hg, respectively. For the detection of GON, the area under the ROC curve was significantly greater for IOPcc compared to GAT. While IOP is unlikely to be an effective diagnostic test, the results of this study suggest that a corneal-compensated IOP may represent a superior test for the evaluation of glaucoma, especially among patients with low to normal IOPs.

## Competing interests

NMR has served as consultant for Allergan and Alcon and has received instrument support from Reichert, Inc.

## Authors' contributions

JRE performed statistical analyses, and helped to draft the manuscript. NMR participated in the design of the study and helped to draft the manuscript. MS conceived of the study, participated in the design of the study and helped to draft the manuscript. All authors read and approved of the final manuscript.

## Pre-publication history

The pre-publication history for this paper can be accessed here:

http://www.biomedcentral.com/1471-2415/12/52/prepub
